# Measuring sustainable development goals performance: How to monitor policy action in the 2030 Agenda implementation?

**DOI:** 10.1016/j.ecolecon.2019.106373

**Published:** 2019-10

**Authors:** Apollonia Miola, Fritz Schiltz

**Affiliations:** a European Commission – Joint Research Centre, Via Enrico Fermi, 2749-I-21027 Ispra, VA, Italy; b European Commission - Joint Research Centr, Via Enrico Fermi, 2749-I-21027 Ispra, VA, Italy; c Economics of Education Research (LEER), Faculty of Economics and Business, KU Leuven, Naamsestraat 69, Leuven 3000, Belgium

## Abstract

Sustainable Development Goals (SDGs) and corresponding targets for 2030 have been adopted by world leaders at the historic UN summit in 2015. Rankings are often constructed in order to hold countries accountable to achieve these targets. This paper illustrates the sensitivity of rankings to the choice of indicators and methodological assumptions by comparing the three most prominent methods using the sample of EU28 countries. The results of our analysis suggest that a country's relative position depends almost entirely on the chosen method and indicators.

## 1 Introduction

The measurement of sustainability has been a topic of fierce debate among researchers, policy makers and other stakeholders (Holden et al., 2014; Costanza et al., 2016). The Adoption of the 2030 Agenda and its Sustainable Development Goals (SDGs), targets and related indicators has further enriched this debate (Allen et al., 2018; Liu et al., 2018).

The 2030 Agenda has been adopted to achieve a better and sustainable future for all. It tries to manage the major challenges we face, recognizing that poverty eradication requires strategies that can work on economic growth by ensuring environmental protection and managing a series of social needs including health, education, and gender equality. Its 17 SDGs and related targets have been designed to be monitored through a set of global indicators adopted together with the 2030 Agenda.

On the one hand the UN Statistical Commission with the High Level Expert Group on SDGs indicators and the various repository agencies (such as UNEP, OECD, etc.) work with different stakeholders for the statistical coverage of the indicators organized around the TIER system, which classifies the UN official SDGs indicators on the basis of their level of methodological development and data coverage.

On the other hand, official statistical institutions, think tanks, and the scientific world contribute to a larger debate on the development of analytics tools for SDGs performance.

We present the state of the art by comparing the three most prominent methods to measure SDGs performance at country level: the Sustainable Development Goals Index developed by the Bertelsmann Stiftung and the Sustainable Development Solutions Network – SDSN (Lafortune et al., 2018; Sachs et al., 2018), the OECD's Distance measure (OECD, 2017), and Progress measures based on Eurostat's report (Eurostat, 2018). We compare and contrast these methods. Our results suggest a strong discrepancy in existing methods. Depending on the chosen indicators and methods applied, countries can receive substantially different relative evaluations. The final aim of this analysis is to highlight crucial weaknesses that should be addressed to provide a context-dependent analysis to measure country SDGs performance.

## 2 Setting the scene

In general terms, we define a performance tool on the basis of its ability to provide an overall assessment of developments in the subject at hand, in a way that can be easily interpreted and communicated to the intended target audience. In the present study, this is based on the evaluation of where countries stand in the different domains with respect to achieving the SDGs in 2030.

The choice of indicators and of their targets can be considered as two central points for the definition of a SDGs performance metric. A pivotal element is the identification of the targets, as many objectives laid down by the 17 SDGs (and their related 169 targets) are not defined in quantitative terms. However, a discussion on how to address the key requirements for selecting the most appropriate set of SDGs indicators is not the objective of our analysis. A SDGs indicator set preferably aligns as closely as possible with the targets put forward in the United Nations 2030 Agenda. The UN Statistical Commission is currently developing an indicator framework for monitoring and reporting the SDGs implementation process globally, acknowledging that different indicators might be appropriate in different contexts. Optimal use of statistical indicators to measure the SDGs is country context-dependent and, in general, there is a trade-off between breadth of coverage and comparability on the one hand and detail and availability of information on the other hand. Some member states have already developed their own set of indicators to assist them in monitoring progress made in their own national SDG implementation policy, typically in the context of activities related to the Voluntary National Reviews. At the same time, the various National Statistical Offices collaborate with the UN Statistical Commission, contributing to the SDG Progress annual report prepared by the General Secretary of the United Nations, which also allows for a comparison of the progress of the various countries at global level.

This paper focuses on the European Union (EU), which has a set of SDGs indicators at the level of the 17 Goals developed by Eurostat through a collaborative process, specifically for the EU context (Appendix A) and based on high quality data. It can be assumed that for the purpose of our analysis the Eurostat SDGs set of indicators are the most appropriate source of data, as they allow a detailed description of the situation in the EU and its Member States, in relation to the 17 SDGs. The set of indicators for EU member states balances comparability across countries with data quality and appropriateness. It should be highlighted that the Eurostat SDGs indicators set is anchored to a high-level scoreboard of EU policies, headline indicators of the Europe 2020 strategy, and indicators included in the social scoreboard for the European Pillar of Social Rights. This choice sometimes made it very difficult to contextualize the indicators in the context of the UN Agenda 2030.

In our analysis we identified the target values available in the UN SDGs framework. Then, for those indicators with no reference in the SDGs framework we searched in other international agreements (such as the Biodiversity Convention for Goal 15) and EU policy documents (such as Europe 2020 or the EU Habitat Directive). Finally, we looked at the peer reviewed and grey literature (such as OECD reports and working Papers). We identified targets for 85 out of 145 Eurostat SDGs indicators.^1^

## 3 Measuring SDG performance

This section reviews three common methods to measure SDGs performance. The range of methods presented here is not comprehensive as our objective is not to review all the existing approaches, yet we aim to highlight crucial weaknesses and identify the main methodological challenges that should be addressed when answering the call for analytical tools to evaluate SDGs performance.

#### 3.1.1 Simple mean

A straightforward approach to constructing a measure of SDG performance is to calculate the mean over all indicators at goal level. As such, all indicators are given an equal weight and the equality of weights applies to all countries – i.e. countries are not allowed to set priorities by weight certain indicators more than others. Before calculating the mean, indicators need to be rescaled in order to account for different unit measures. For example, the average of the employment rate (in percentages, between 0 and 100) and the GDP per capita (in $) will be skewed towards the latter measure. After rescaling all variables, all values are expressed relatively, and the unit of measurement does not matter anymore. The aggregation at goal level can be noted as follows:

$$I_{\mathrm{ij}}^{M}=\sum_{k=1}^{N_{\mathrm{ij}}}\frac{1}{N_{\mathrm{ij}}}\frac{x_{\mathrm{ijk}}-\min_{\mathrm{jk}}}{\max_{\mathrm{jk}}-\min_{\mathrm{jk}}}$$

where *N*_*ij*_ is the number of indicators for SDG *j* for which country *i* has data and *x*_*ijk*_ denotes the score for indicator *k* (within SDG *j*) for country *i.* Subtracting the minimum value across countries for indicator *k* (*min*_*jk*_), and dividing this difference by the range (*max*_*jk*_ − *min*_*jk*_) results in a rescaled value between 0 and 1. This rescaled value is then aggregated with equal weights over all indicators *k* within SDG *j* to obtain a measure of performance for country *i*.

This simple mean approach is adopted by the Bertelsmann Stiftung and SDSN when constructing the Bertelsmann Index (BI), both at goal level and overall. The construction of the BI comprises three main steps, described in more detail in their technical report (Lafortune et al., 2018). Using different data sources (WB, OECD, IMF, UN …) the BI results from (Holden et al., 2014) censoring outliers, (Costanza et al., 2016) rescaling and (Allen et al., 2018) weighting the different indicators. The resulting index measures the relative performance of countries to all countries included in the assessment. Low, medium and high indicator values are also assigned to develop a dashboard assessing country progress for each indicator.

#### 3.1.2 Distance measure

An alternative approach to measuring SDG performance is provided by the OECD's distance measure (OECD, 2017). Their approach constitutes of calculating the standardized distance to a specified target for each indicator. This is done by dividing the absolute distance of a country to the target and dividing this number by the standard deviation in all countries' performance on the evaluated indicator. Values below 0 (country surpassed the target) are set to 0. As such, the relative performance of a country will be strongly dependent on which countries were included to compute the standard deviation. Although other data sources were also used, the main input for the analyses was OECD data. The computation of the distance measure for country *i* with respect to SDG *j* can be denoted as follows:

$$I_{\mathrm{ij}}^{D}=\sum_{k=1}^{N_{\mathrm{ij}}}\frac{1}{N_{\mathrm{ij}}}\max\left(\frac{T_{\mathrm{jk}}-x_{\mathrm{ijkt}}}{{\mathrm{SD}}_{\mathrm{jk}}},0\right)$$

where *T*_*jk*_ is the target for indicator *k* in SDG *j*, *x*_*ijk*_ is the last available observation *t* for country *i* with respect to indicator *k* in goal *j*. In order to obtain positive values by subtracting a country's value and the target for indicator *k*, all values were transformed such that higher values are preferred. If a country exceeds the target in the latest available year, its distance is set to 0. Once distance measures are obtained for each indicator *k*, a country's score for SDG *j* can be computed by calculating the simple average of all distances.

#### 3.1.3 Progress measure

A third measure of SDG performance is based on the monitoring report by Eurostat on the progress towards the SDGs in an EU context (2018). We constructed a 2030 value for each country assuming ‘business-as-usual’ (BAU). The BAU growth rate was calculated by extracting the first and last observation for each indicator and by linearly interpolating the end values in 2030. Resulting values for 2030 were aggregated within goals by feature scaling and equal weighting. The advantages of this approach are twofold. First, many more indicators can be included as no targets are required. Second, the use of Eurostat data allows an assessment of SDG performance that accounts for the specific EU context. Formally, this can be written as:

$$I_{\mathrm{ij}}^{P}=\sum_{k=1}^{N_{\mathrm{ij}}}\frac{1}{N_{\mathrm{ij}}}\frac{x_{\mathrm{ijk}}^{t^{1}}-x_{\mathrm{ijk}}^{t^{0}}}{t^{1}-t^{0}}\left(2030-t^{1}\right)+x_{\mathrm{ijk}}^{t^{1}}$$

where *x*_*ijk*_^*t*^1^^ (*x*_*ijk*_^*t*^0^^) is the latest (first) observation of country *i* with respect to indicator *k* in goal *j*. The difference between 2030 and the latest year of observation (*t*^1^) determines the value to be added to the latest observation *x*_*ijk*_^*t*^1^^ to get the interpolated value for 2030 – assuming a linear trend continues. In order to obtain a performance measure at goal level, progress measures were averaged over all indicators after rescaling values between 0 and 1, similar to the aggregation step done for the simple mean and the Bertelsmann Index.

## 4 Comparison of existing methods and indicators

To compare the three methods presented above, we constructed mean values, distance measures and progress measures for all goals using Eurostat SDGs set of indicators (2018) for EU28 countries. To contrast these results, we constructed rankings based on the Bertelsmann Index by collecting data for each goal and each EU28 country (Lafortune et al., 2018; Sachs et al., 2018). In contrast, we cannot use the OECD's distance measure as it is not available for all EU28 countries. Therefore, we applied their methodology to Eurostat indicators with identified policy targets (marked in green in Appendix A). In addition, we calculated the average score (as in Section 3.1.1) using the same subset of indicators with targets. Table 1 summarizes the five SDGs measures which were calculated, the corresponding number of indicators, the data source, and the methods used. All measures of SDGs performance, apart from the BI, were obtained using the statistical software R and Eurostat SDGs set of indicators.

**Table 1:** Comparison of SDG measures.

	Bertelsmann Index	Mean	Progress	Mean (T)	Distance (T)
Method	Simple mean	Simple mean	Progress	Simple mean	Distance
Number of Indicators	88	145	145	85	85
Main data source	Multiple, as reported in Sachs et al. (2018) - p. 46.	Eurostat SDGs set of indicators (2018) -Appendix A	Eurostat SDGs set of indicators (2018) with identified targets – marked in green in Appendix A	Eurostat SDGs set of indicators (2018) with identified targets – marked in green in Appendix A	Eurostat SDGs set of indicators (2018) with identified targets – marked in green in Appendix A

**Fig. 1:**
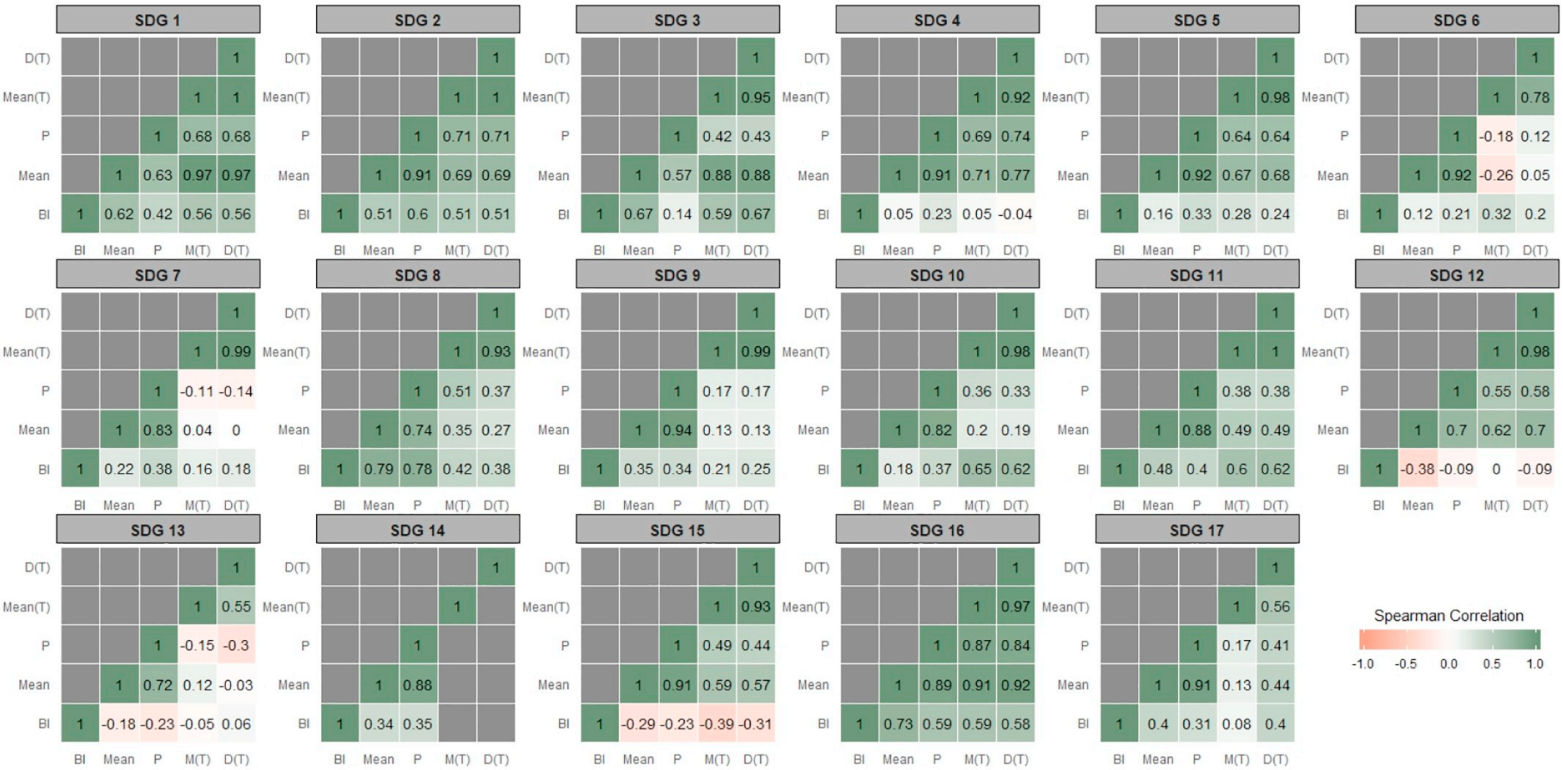
Rank order correlations by SDG. *Note*: This figure displays rank order correlations for different combinations of SDG measures, obtained using different indicators and methodologies: “BI” (Bertelsmann Index), “Mean” (Simple mean of all Eurostat indicators), “P” (progress measure using 2030 predictions for all Eurostat indicators), “Mean (T)” (Simple mean using Eurostat indicators where EU targets could be identified, Appendix A), “D (T)” (Distance measure using Eurostat indicators where EU targets could be identified, Appendix A).

Using all five measures, we computed rank order correlations (Spearman) to illustrate the sensitivity of rankings to the chosen set of indicators and the chosen methodology. Fig. 1 displays the rank order correlations for each single SDG. Overall, most rankings are consistent across methods and goals but some specific cases require further attention. For example, rankings obtained for SDG 1 are roughly independent of the method used. However, correlations are lowest between the Bertelsmann rankings (BI) and others, suggesting the importance of the chosen set of indicators. In contrast, the chosen methodology to rank countries does not appear to be crucial for SDG 1. That is, using a simple mean, a distance measure or a progress measure results in rankings that are highly inter-correlated (ranging between 0.68 and 1), holding the set of indicators constant.

Analyzing the rank order correlations for all other goals, the use of indicators differing from the Eurostat data by the BI seems to result in inconsistent rankings for SDG 12, SDG 13 and SDG 15. In general, changing the set of indicators appears to affect the ranking of EU28 countries more than changing the chosen method (correlations are typically lowest on the bottom row of all matrices).

This motivates the use of Eurostat SDGs set of indicators when ranking EU member states in terms of SDG performance, as these indicators are able to capture the EU context more adequately. Second, restricting the set of indicators to those where official targets could be identified does not seem to affect rankings substantially. This can be seen by the relatively strong correlations between ‘Mean’ (145 indicators) and ‘Mean (T)’ (85 indicators). Exceptions include SDG 4, SDG 12 and SDG 17. For SDG 13 and SDG 15, there is even a negative correlation between both. This implies that the ranking can change completely for these goals when a subset of the available indicators is used. As such, it can be recommended to use all available indicators. Third, choosing the simple mean or the distance measure to aggregate performance at goal level does not seem to affect relative rankings of EU28 countries. Most rank order correlations are close to 1 and even equal to 1 (identical ranks obtained by both methods) for SDG 1, SDG 2, and SDG 11.

**Fig. 2:**
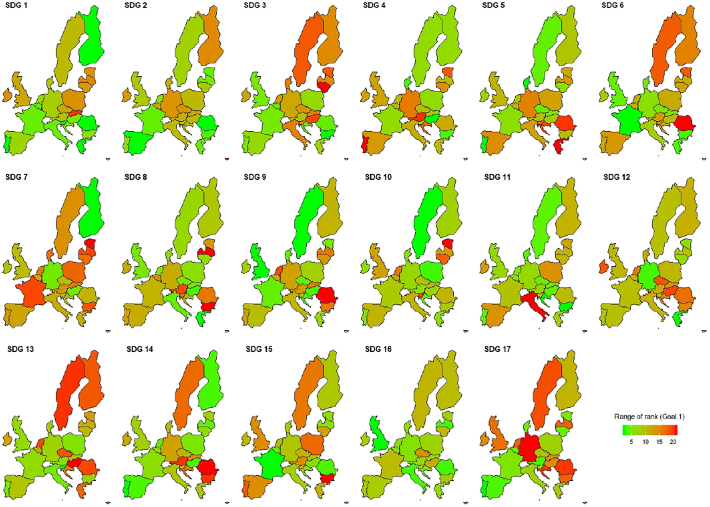
Range of rankings by SDG. *Note*: This figure displays the difference between the maximum and minimum rank (range) assigned to each country using different indicators and methodologies: Bertelsmann Index, simple mean of all Eurostat indicators, Progress measure using 2030 predictions for all Eurostat indicators, simple mean using Eurostat indicators where EU targets could be identified (Appendix A), and a Distance measure using Eurostat indicators where EU targets could be identified (Appendix A).

As a final remark, Fig. 1, Fig. 2 also indicate some discrepancy between rankings resulting from the Progress measure and others. This can again be explained by the different set of indicators used when constructing the Distance measure and the Progress measure (see Table 1). When comparing only the methods while holding the number of indicators constant (i.e. “Mean” and “P”), there is an overall strong correlation between rankings. Once again, our findings suggest that the choice of indicators is of paramount importance, while the rank changes due to the chosen method are less pronounced, *on average*. However, methodological choices can result in major implications for *individual countries* in terms of their SDG ranking.

When we compare the change in rankings for individual countries, a more nuanced picture emerges. Fig. 2 maps the range of rankings for each single SDG. This range was computed by subtracting a country's lowest rank from its highest rank, obtained from each of the five methods in Table 1. Green countries indicate a relatively small change in ranking when indicators and methods are changed. Red countries indicate a strong sensitivity to these choices. For example, Slovakia and Luxembourg change their ranking within EU28 countries by 15 to 20 places depending on the chosen methodology and set of indicators while other countries, e.g. Italy and Portugal, receive very consistent rankings. Two results become apparent when analyzing Fig. 2. First, some goals result in more robust rankings (e.g. SDG 16) while other goals are very sensitive to methodological choices (e.g. SDG 7). Second, some countries change their ranking a lot for certain goals, while they retain consistently their relative positions for other goals. For example, Sweden receives very consistent ranks for SDG 5 while the choice between Simple mean, Distance, Progress or BI matters a lot for SDG 13 in Sweden.

## 5 Discussion

In the previous section, we highlighted how relative rankings of EU member states are strongly dependent on the choice of methods, and even more so on the choice of indicators. The question remains why context seems to be such an important factor in determining to what extent this particular choice matters. For example, for certain SDGs, this choice did not seem to be crucial (e.g. SDG 1 or SDG 2 in Fig. 2), while in some countries only little variation could be observed across methods and indicators – and even across SDGs (e.g. Italy). One explanation that comes to mind is that countries who consistently under- or over-perform in all indicators might not be affected by the choice of weights, implicit in the chosen method. However, the example of Italy opposes this hypothesis. Although Italy's performance on many indicators is only moderate, it is far from the bottom performer, let alone the bottom performer on all indicators. In our view, there are three hypotheses that might explain the context-dependency of SDGs performance measures. We invite researchers to challenge these in future research and, in so doing, contribute to more robust indicators.

First, one could argue that homogeneous SDGs are captured by more comparable indicators. Looking at Appendix A, it can be seen that indicators for SDG 16 are defined in a narrow sense (with homicide, violence, and sexual violence being strong correlates), whereas the indicator composition of SDG 6 can be considered quite dispersed (with indicators on population having a bath to biochemical oxygen demand in rivers). We do not claim that more SDGs will be needed to ensure more narrow definitions, yet we conclude from our findings that some SDGs are monitored using a somewhat loosely defined indicator set, complicating the comparison of countries as priorities – and hence weights - can be misaligned. We see it as an interesting avenue for future research to evaluate the statistical consistency of SDGs by validating if indicators that are being grouped together actually measure one underlying concept. This could be done by factor, principal component, and reliability analysis, using different data sets, extending the application at hand to EU countries.

Second, differences in policy priorities between countries could exacerbate the importance of choosing a method or indicators. For example, countries that deliberately progress faster in specific indicators at the cost of others will be at a disadvantage when each indicator is given the same weight. It would therefore be an interesting exercise to repeat our analysis focusing solely on countries that explicitly claim to place equal weights on subcomponents of each SDG, and vice versa.

Finally, when targets are missing, the indicators used to obtain a ranking will differ across methods. This follows directly from the fact that not all approaches require targets, yet having targets facilitates the measurement of actually achieving SDGs in 2030 (see Section 3). For example, SDG 13 “Climate Action” consists of 11 indicators, while quantifiable targets could only be identified for 3 of them. It is therefore of major importance that policy agree upon a set of tangible targets, making it easier to benchmark countries to identify best practices, while holding low performers accountable in a credible way. In doing so, policy makers can contribute to the achievement of the SDGs in 2030.

Note that a combination of the above serves as an additional explanation. When the consistency between indicators is not particularly high and a national government decides to prioritize a specific indicator, performance evaluations could be strongly dependent on method choice when a target for this indicator cannot be identified. Once again, this stresses the importance to agree upon a common framework for quantifiable SDG targets.

## 6 Conclusion

The complexity and the richness of the current debate on how to measure countries SDGs performance is both technical and political. This characteristic makes it difficult to disentangle between a jungle of indices and indicators to measure SDG performance.

On the technical point of view, the presence of plurality of frameworks, possible interpretations and the selection of indicator variables preclude a consensus on a “right” or “objective” method to measure SDGs performance. This paper highlights a strong discrepancy in three main existing methods. Our results suggest that a country's relative position depends almost entirely on the chosen method and indicators. For example, in our analysis rank order correlations are sometimes close to 0 or even negative, while individual countries can be either at the top or at the bottom of the EU28 ranking, depending on assumptions made. Naturally, these differences were to be expected as they measure different things.

A different set of indicators implies different priorities. Therefore, we argued that country context dependent indicators should be used as they provide a more consistent data source to evaluate performance of a country. Nonetheless, our results also indicated that rankings can be sensitive to the chosen aggregation method, even when the same indicators are used. This highlights the importance of imposing assumptions when aggregating at goal level. For example, averaging indicators into one composite score at goal level is different from making linear interpolations to 2030 when using Progress measures. The former assumes equal weighting while the latter adds the additional assumption of linearity when making predictions. Each choice of methodology, and hence a choice of assumptions, affects weighting priorities, which in turn can have major implications for relative rankings (Booysen, 2002).

We also highlight an important gap in each of the analyzed methods. None of such methods considers interlinkages. Although this topic is extremely complex (Miola et al., 2019) the holistic nature of the 2030 Agenda makes it a key element of every SDGs implementation policy, thus, emphasizing even more the contextualization of choice of the SDGs performance method.

On the political point of view, the existence of multiple, and in principal equally justifiable, indicator sets and the possibility of conflicting results in aggregating them creates a complex situation. On the one hand, a clear message needs to be communicated. On the other hand, an overly narrow focus on one single analysis can create confusion as the public is confronted with different results. In this context, the strictly political significance of the choice of indicators and methods to monitor the SDGs performance is the central element. The lack of this kind of considerations could be considered as the major weakness of the existing methods. In our opinion ranking countries is not a suited approach to the 2030 Agenda since the search for the best performer is not the purpose of the 2030 Agenda. In some countries the process of implementing the SDGs framework could be more important than the final result in terms of performance. We, then, consider the SDGs performance analysis as a tool informing how much effort is needed to achieve SDGs at country level. It is therefore up to each State to define country specific targets and, then, the choice of the most suited indicators. Moreover, monitoring progress at the level of a single country compared to other countries at global level can be done within the context of the SDG Progress annual report prepared by the General Secretary of the United Nations.

## Figures and Tables

**Table:**
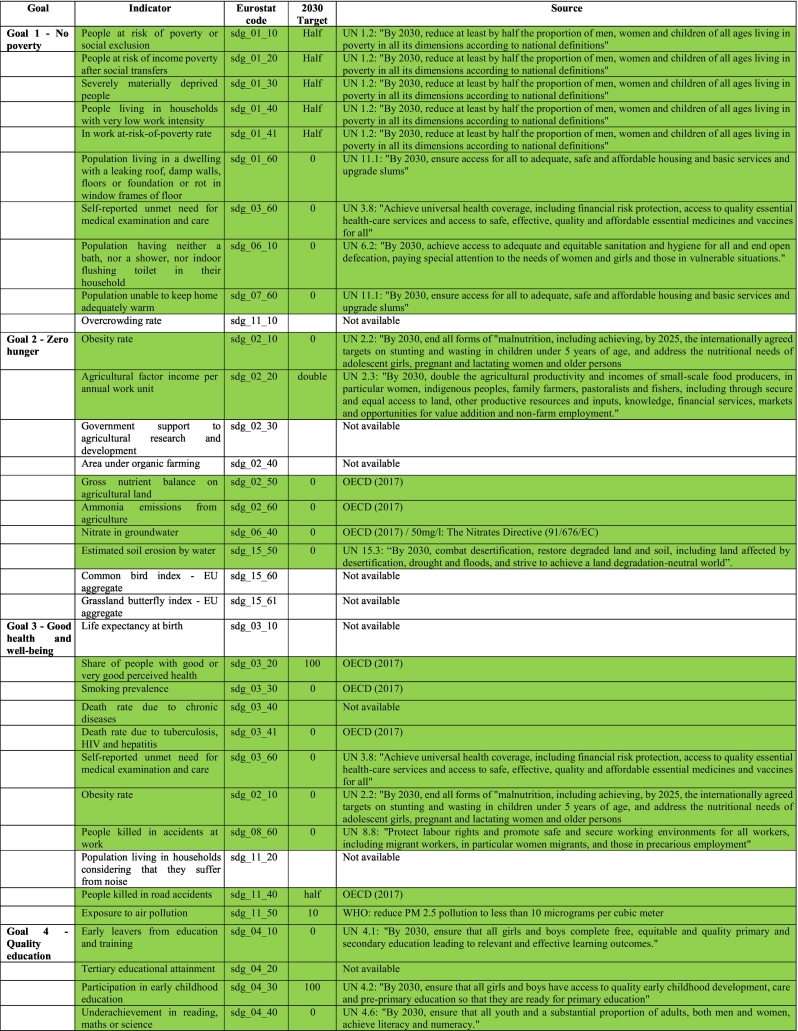

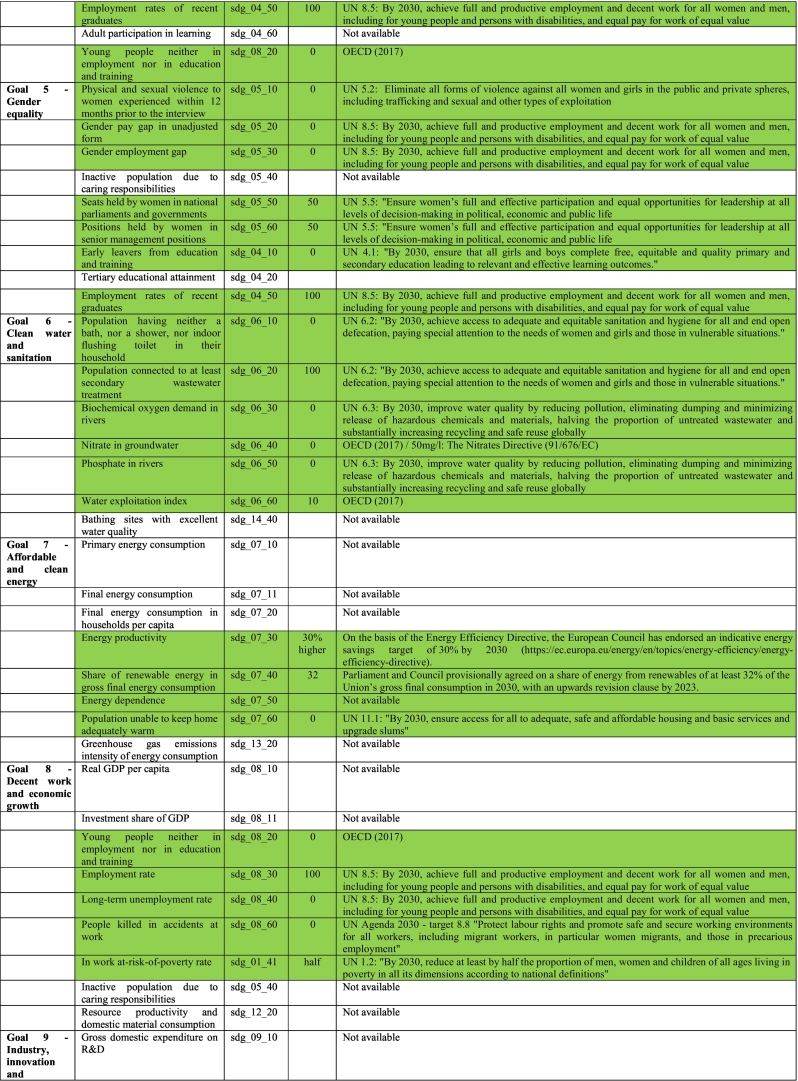

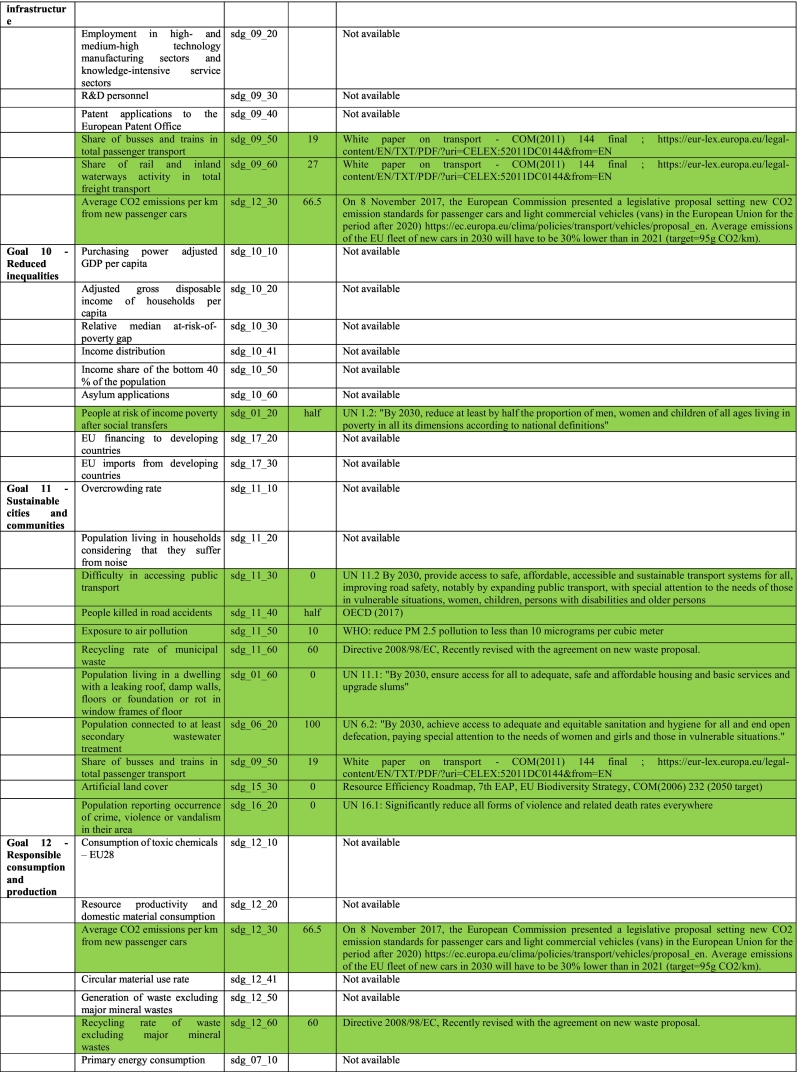

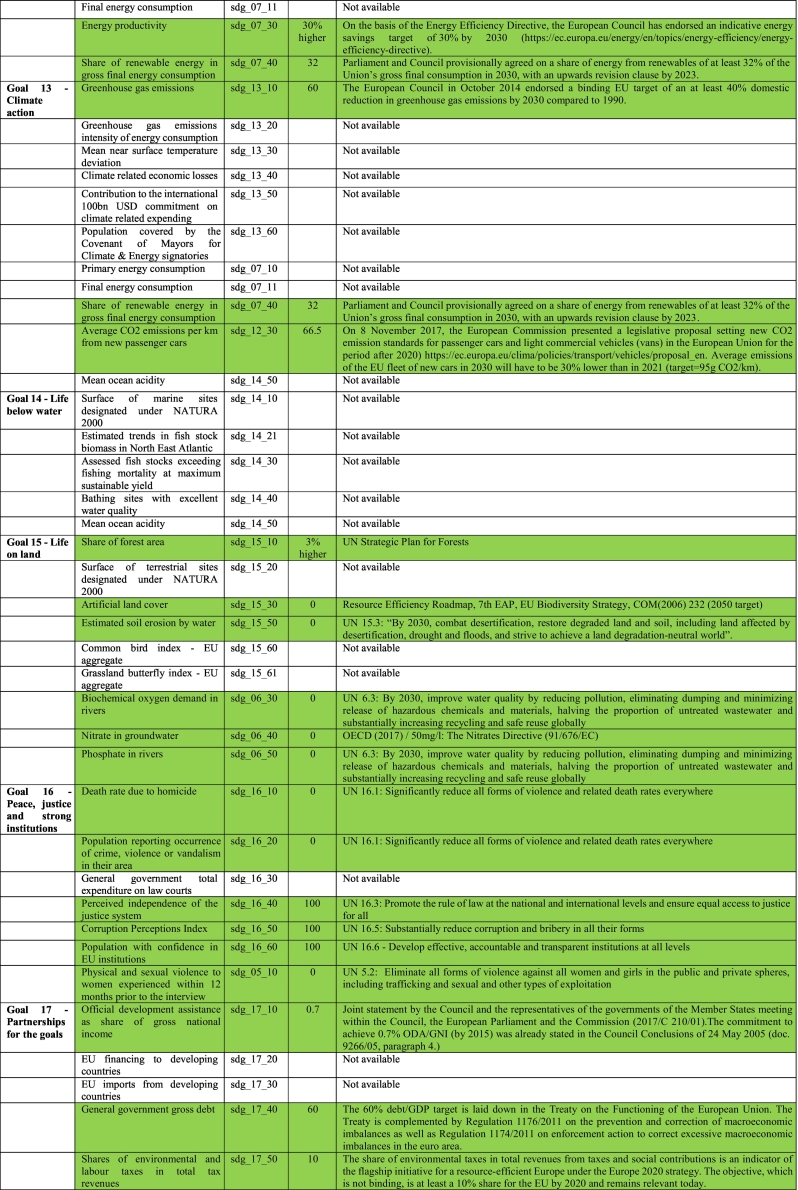


## Footnotes

1 Appendix A presents the list of Eurostat SDGs indicators for 2018, the list of identified targets and the rationale behind them. Indicators where a target could be identified are marked in green.
